# Editorial to “pseudo‐slow‐fast atrioventricular nodal reentrant tachycardia: Is the fast pathway a criminal or innocent bystander?”

**DOI:** 10.1002/joa3.12978

**Published:** 2023-12-13

**Authors:** Yoshiaki Mizutani, Satoshi Yanagisawa, Yasuya Inden

**Affiliations:** ^1^ Department of Cardiology Yokkaichi Municipal Hospital Yokkaichi Mie Japan; ^2^ Department of Cardiology Nagoya University Graduate School of Medicine Nagoya Aichi Japan

Atrioventricular nodal reentrant tachycardia (AVNRT) represents one of the most common regular supraventricular tachycardias (SVTs), and catheter ablation is an effective treatment with a high success rate for improving the quality of life of symptomatic patients. However, the exact mechanism and detailed characteristics of AVNRT are yet to be clarified.

In this issue of the journal, Hirata et al.^1^ reported a very interesting and rare case of “pseudo‐slow‐fast AVNRT,” which suggests a slow‐slow AVNRT mimicking a slow‐fast AVNRT. The authors discussed the possibility of all types of narrow QRS tachycardias in detail through an electrophysiological study. They presented two different sequences of narrow QRS tachycardias (SVT1 and 2) in the same patient: SVT1 was characterized by sustained tachycardia with VA block and sinus rhythm (SR) during the tachycardia resetting His and ventricular components but not the tachycardia. First, the possibility of intra‐Hisian re‐entry, upper septal‐type idiopathic left ventricular tachycardia, and junctional tachycardia was excluded. The resetting of His and ventricular components, but not the tachycardia, indicated that the antegrade fast pathway was a bystander, and the possibility of orthodromic reciprocating tachycardia with concealed nodoventricular or nodofascicular pathways was excluded. The earliest atrial activation site (EAAS) was the proximal coronary sinus (CS). Therefore, the final diagnosis of SVT1 was slow‐slow AVNRT with an upper common pathway. After isoproterenol administration, SVT2 occurred. The EAAS of SVT2 was the His region, which seemed to indicate a different diagnosis from that of SVT1. Premature ventricular extrastimuli in the His refractory period did not reset the tachycardia, while earlier ventricular extrastimuli reset the tachycardia without a sequence change. These findings are consistent with the typical phenomenon of a slow‐fast AVNRT. However, following further earlier ventricular extrastimuli with a shorter coupling interval, the EAAS changed to the CS proximal with jump up and no resetting of the tachycardia. These results indicated that the retrograde fast pathway was a bystander. Therefore, the SVT2 was also a slow‐slow AVNRT, indicating that SVT1 and SVT2 were identical tachycardias.

The conclusions of this study can be supported in several ways. An additional investigation to measure the longer coupling interval of a single extra stimulus from the proximal CS region than near the His bundle site to reset SVT2 may further support that SVT2 is a slow‐slow AVNRT.^2^ In addition, it may have been better to test reproducibility by gradually shifting the timing of the ventricular extrastimuli to exclude the possibility that the early ventricular extrastimuli accidentally changed the retrograde conduction from the fast pathway to the slow pathway.

Nevertheless, the findings of the study by Hirata et al. provide valuable information that will help specialists in electrophysiology and arrhythmia to expand their understanding of the underlying mechanisms and characteristics of AVNRT. In particular, an early ventricular extrastimuli during SVT can be helpful in diagnosing whether the true circuit of SVT represented as a slow‐fast AVNRT, such as SVT2, is correct. Previous reports have indicated that the fast pathway does not participate in fast‐slow AVNRT.^3^ However, to the best of our knowledge, no reports have demonstrated that SVT is normally considered a slow‐fast AVNRT but is in fact a slow‐slow AVNRT with a bystander fast pathway. As the changed electrical distance of the EAAS between the components in the CS proximal and His bundle region was quite minimal on the intracardiac electrogram in this case, careful observation of the retrograde jump up after early ventricular extrastimuli might be essential and useful for identifying mimicked slow‐fast AVNRTs. Although a question is raised regarding how frequently such a mimicked AVNRT is involved in a typical slow‐fast AVNRT, it is possible that a certain number of “pseudo‐slow‐fast AVNRTs” are actually mixed in with those diagnosed as slow‐fast AVNRTs. Further systematic evaluations of this rare phenomenon using large‐scale samples are required.

## FUNDING INFORMATION

This research did not receive any specific grant from funding agencies in the public, commercial, or not‐for‐profit sectors.

## CONFLICT OF INTEREST STATEMENT

The authors declare no conflicts of interest.
